# Inhibition of LATS kinases reduces tumorigenicity and increases the sensitivity of human chronic myelogenous leukemia cells to imatinib

**DOI:** 10.1038/s41598-024-54728-z

**Published:** 2024-02-18

**Authors:** Phatchanat Klaihmon, Chanchao Lorthongpanich, Pakpoom Kheolamai, Wannachai Saisaard, Surapol Issaragrisil

**Affiliations:** 1 Siriraj Center of Excellence for Stem Cell Research, Faculty of Medicine Siriraj Hospital, Mahidol University, Bangkok, Thailand; 2 Blood Products and Cellular Immunotherapy Research Group, Faculty of Medicine Siriraj Hospital, Mahidol University, Bangkok, 10700 Thailand; 3 Center of Excellence in Stem Cell Research and Innovations, Division of Cell Biology, Faculty of Medicine, Thammasat University, Pathumthani, Thailand; 4 Division of Hematology, Department of Medicine, Faculty of Medicine Siriraj Hospital, Mahidol University, Bangkok, Thailand

**Keywords:** Chronic myelogenous leukemia, Hippo pathway, LATS kinase, Apoptosis, Imatinib, Cancer, Cell biology, Molecular biology

## Abstract

Chronic myelogenous leukemia (CML) is a clonal hematologic malignancy of the myeloid lineage caused by the oncogenic BCR/ABL fusion protein that promotes CML cell proliferation and protects them against drug-induced apoptosis. In this study, we determine LATS1 and LATS2 expression in CML cells derived from patients who are resistant to imatinib (IM) treatment. Significant upregulation of LATS1 and LATS2 was found in these CML patients compared to healthy donors. To further explore whether the expression of LATS1/2 contributes to the IM-resistant phenotype, IM-resistant CML cell lines generated by culturing CML-derived erythroblastic K562 cells in increasing concentrations of IM were used as in vitro models. Up-regulation of LATS1 and LATS2 was observed in IM-resistant K562 cells. Reduction of LATS using either Lats-IN-1 (TRULI), a specific LATS inhibitor, or shRNA targeting LATS1/2 significantly reduced clonogenicity, increased apoptosis and induced differentiation of K562 cells to late-stage erythroid cells. Furthermore, depletion of LATS1 and LATS2 also increased the sensitivity of K562 cells to IM. Taken together, our results suggest that LATS could be one of the key factors contributing to the rapid proliferation, reduced apoptosis, and IM resistance of CML cells. Targeting LATS could be a promising treatment to enhance the therapeutic effect of a conventional BCR/ABL tyrosine kinase inhibitor such as IM.

## Introduction

Chronic myelogenous leukemia (CML) is a hematopoietic malignancy caused by reciprocal translocation of chromosomes 9 and 22 in myeloid cells that generate an oncogenic fusion protein BCR/ABL^1,2^. BCR/ABL, a constitutively activated tyrosine kinase, activates several intracellular signaling pathways that promote the growth, proliferation, and survival of CML cells^3^. Imatinib (IM) is a first-line drug for patients with CML that inhibits BCR/ABL phosphorylation and prevents its cancer-promoting signals^4^. Despite its effectiveness, some CML patients, particularly those with the blast-crisis phase of the disease, did not respond well to the drug. This resistance to imatinib could be caused by mutations in the ABL kinase domain that reduce drug affinity or alterations in other downstream signaling pathways that increase CML cell survival^5^. To overcome this limitation, it is essential to find additional drug targets that could be used to improve the effectiveness of IM in patients with CML.

The Hippo signaling pathway was first identified in *Drosophila melanogaster*^6,7^ and has since been shown to regulate cell growth, proliferation, survival, and differentiation in many organisms, including humans^8–10^. In humans, the pathway consists of several key components, including STE20-like kinase 1/2 (MST1/2), Salvador homologue 1 Yes-associated protein 1 (YAP), WW domain-containing transcription regulator 1 (TAZ), and transcriptional enhanced associated domain (TEAD). YAP/TAZ/TEAD are transcriptional coactivators that regulate the expression of many Hippo signaling target genes that control cell proliferation, apoptosis, and stem cell self-renewal in many human tissues^11,12^. Aberrant regulation of the Hippo pathway has been shown to play an important role in the pathogenesis of various epithelial and hematologic malignancies, including CML^13^. Although the expression levels of *LATS* and *TAZ*, together with the Aurora kinase genes, have been shown to correlate with the level of IM resistance in CML patients, the mechanism underlying this correlation has not yet been characterized^14^. Therefore, this study aims to study the roles of the Hippo signaling pathway in human CML cells using a pharmacological treatment and genetic manipulation to inhibit *LATS,* an important component of Hippo signaling, in a human CML cell line, K562. The effects of LATS inhibition on survival, proliferation, erythroid differentiation, ROS production, and imatinib resistance of K562 cells were investigated.

## Results

### The LATS1 and LATS2 were highly expressed in leukemic cells from CML patients

**Figure 1:**
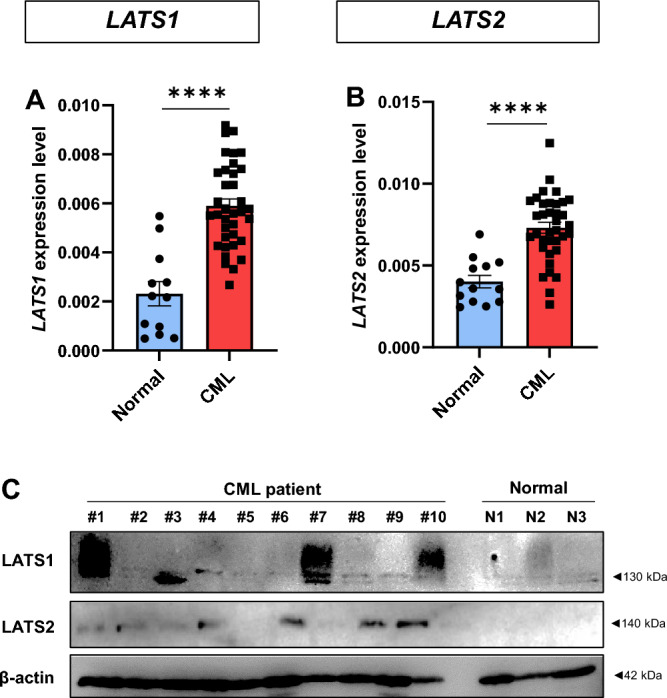
High expression of the LATS family in CML samples. Comparison of mRNA levels of *LATS1* (**A**) and *LATS2* (**B**) in normal subjects versus CML patients. Western blot analysis of LATS1 and LATS2 in mononuclear cells obtained from normal individuals and CML patients (**C**). ****p < 0.0001. Each dot represents each healthy subject and CML patient.

LATS1 and LATS2 have previously been shown to be highly expressed in CML cells^14^. To determine whether our Thai CML patients who are resistant to IM treatment also had high expression of *LATS*, we determined the expression of *LATS1* and *LATS2* transcripts in 60 adult CML patients diagnosed with the presence of the Philadelphia chromosome and the BCR–ABL fusion gene compared to healthy donors. The levels of *LATS1* and *LATS2* expression in CML patients were significantly higher than those of healthy donors (Fig. 1A–B). Consistent with mRNA levels, higher levels of LATS1 and LATS2 proteins were observed in 10 randomly selected IM-resistant CML patients compared to healthy donors (Fig. 1C). These data confirm that LATS1 and LATS2 are highly expressed in IM-resistant CML patient at both mRNA and protein levels and up-regulation of LATS1 and LATS2 might be involved, at least in part, in the pathogenesis of CML.

### Reduction of LATS1/2 induced apoptosis and induced erythroid differentiation of CML cells

**Figure 2:**
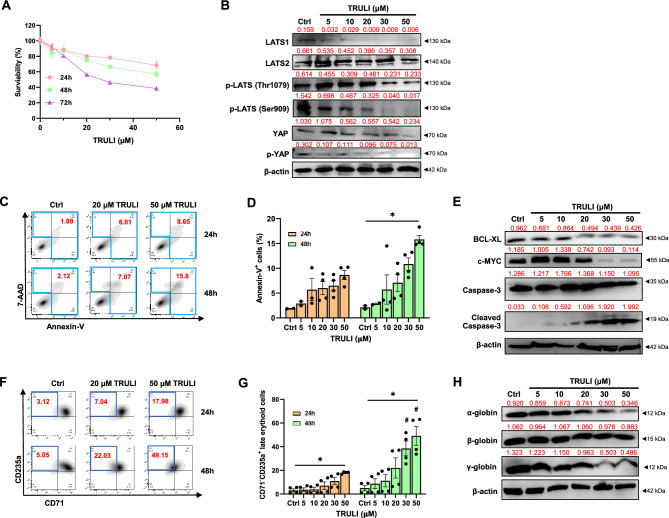
TRULI treatment induces erythroid differentiation and apoptosis of CML-derived cells. Dose–response curve of TRULI-treated K562 cells at different concentrations for 72 h performed by the MTT assay (**A**). Western blot analysis of Hippo components from TRULI-treated K562 cells for 48 h (**B**). Flow cytometric (FACS) analysis of cell death determined by annexin-V/7-AAD staining of TRULI-treated K562 cells (**C** and **D**). Protein levels of apoptosis (caspase-3) and anti-apoptosis (BCL-XL and c-MYC) in TRULI-exposed K562 cells for 48 h (**E**). FACS analysis of the erythroid differentiation marker (CD235a/CD71) in TRULI treated K562 cells (**F** and **G**). Western blot analysis of human globin protein levels in TRULI treated K562 cells for 48 h (**H**). *p < 0.05; ^#^*p* < 0.05 (vs 24 h). Each dot represents the mean of each independent experiment. Band intensities of Western blot analysis normalized by β-actin were labeled above each lane.

Due to the limited number of patient-derived CML cells, human erythroblastic CML K562 cells were used to study the biological effects of LATS1/2 on the survival and differentiation of human CML cells in vitro. To inhibit LATS1/2 expression, K562 cells were treated with LATS1/2 inhibitor (TRULI) for 3 days before being harvested for further experiments. The result shows that TRULI reduced the viability of K562 cells in a dose- and time-dependent manner; its half-maximal inhibitory concentration (IC_50_) at 24, 48, and 72 h is 54.12, 29.09 and 29.47 µM, respectively (Fig. 2A). As expected, TRULI profoundly reduced the level of LATS1 and phosphorylated LATS (p-LATS), an active form of LATS, as well as the levels of YAP and its phosphorylated form (p-YAP, which are downstream targets of p-LATS and the main transcription factors in the Hippo signaling pathway), in a dose-dependent manner (Fig. 2B).Reduction of LATS1/2 by TRULI also increased the percentages of apoptotic K562 cells in a dose- and time-dependent manner (Fig. 2C and D). Consistent with its apoptotic inducting effect, TRULI reduced the expression levels of anti-apoptotic proteins, BCL-XL and c-MYC, while increasing the level of apoptosis-associated cleaved caspase-3 protein (Fig. 2E). Furthermore, TRULI also induced the differentiation of K562 cells to late-stage erythroid cells (CD71^−^/CD235a^+^ cells) in a dose- and time-dependent manner (Fig. 2F and G). However, this differentiation toward late erythroid cells was not accompanied by increased levels of globin proteins (Fig. 2H), which could be resulted from apoptosis-mediated protein degradation, especially from high doses of TRULI. These results suggest that pharmacological inhibition of LATS reduces proliferation, promotes spontaneous differentiation to late-stage erythroid cells, and increases apoptosis of K562 cells.

### LAT1/2 inhibitor inhibited colony formation, migration, and ROS production in K562 cells

**Figure 3:**
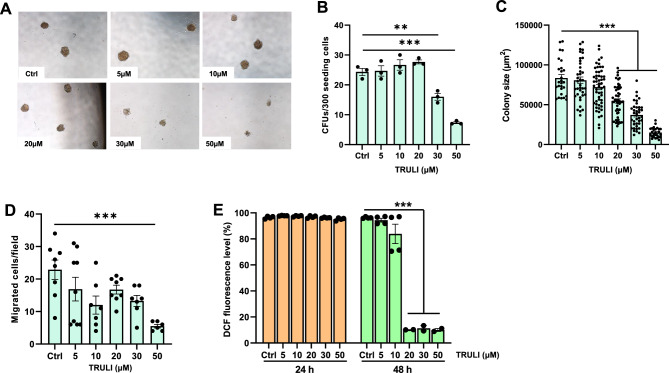
TRULI-treated K562 cells decrease clonogenicity, migratory capacity, and ROS formation. Representative photographs of the colony-forming assay of K562 cells exposed to different concentrations of TRULI (**A**). Numbers and size of leukemic colonies of TRULI treated K562 cells (**B** and **C**). Numbers of migrated K562 cells treated with TRULI for 48 h performed by Transwell migration assay (**D**). Intracellular ROS levels in TRULI-treated K562 cells detected by FACS-based DCFDA/H2DCFDA assay (**E**). **p < 0.01; ***p < 0.001. Each dot represents the mean of each independent experiment, except that a dot in Fig. 3C represents an individual colony.

To further determine the biological effects of LATS1/2 on other aspects of CML cell property, TRULI-treated K562 cells were subjected to clonogenicity, migration, and reactive oxygen species (ROS) detection assays. The results show that TRULI, at a concentration of 30 uM or greater, significantly reduces the number of K562 colonies in the colony formation assay (Fig. 3A and B). Additionally, the size of the derived colonies was also reduced in a dose-dependent manner (Fig. 3C). These results suggest that inhibition of LATS1/2 by TRULI could inhibit colony-forming capacity and proliferation of CML cells. Furthermore, TRULI also significantly reduced migration (Fig. 3D), and inhibited ROS production, a hallmark of cancer metabolism, in K562 cells at 48 h (Fig. 3E). Taken together, these results suggest that inhibition of LATS1/2 efficiently reduces the tumorigenic properties and aggressiveness of CML cells.

### The LATS inhibitor reversed the IM resistant phenotype of CML cells

**Figure 4:**
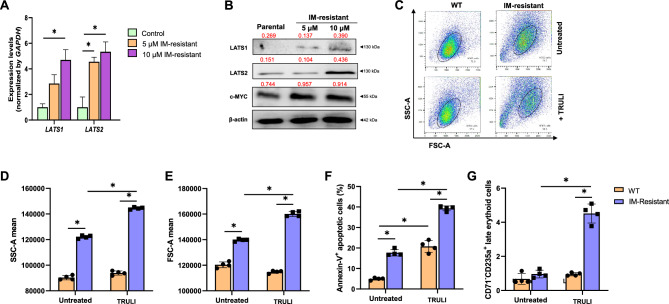
TRULI treatment ameliorates IM-resistance. Expressions of genes and proteins of the LATS family in IM-resistant K562 cells (**A** and **B**). Representative dot plot of 10 µM IM-resistant K562 cells in the absence or presence of TRULI at 20 µM for 48 h (**C**). Geomean of side- and forward-scatter of IM-resistant K562 cells treated with TRULI compared to untreated K562 cells (**D** and **E**). Percentages of the apoptotic and late erythroid stage of IM-resistant K562 cells in the absence or presence of TRULI at 20 µM for 48 h (**F** and **G**). **p < 0.05. Each dot represents the mean of each independent experiment. Band intensities of Western blotting analysis normalized by β-actin were labeled above each lane.

To further investigate whether the levels of LATS1 and LATS2 in K562 cells are affected by imatinib treatment, cells were cultured with increasing concentrations of IM (for more than 10 cycles of treatment) to establish K562 cells that were resistant to various concentrations of IM. The results show that the expression of *LATS1* and *LATS2* transcripts and proteins are indeed up-regulated in imatinib-resistant K562 cells in a dose-dependent manner (Fig. 4A and B). The increase in LATS1 and LATS2 proteins in these IM resistant K562 cells was accompanied by an increase in the level of the c-MYC protein, a well-known target of Hippo signaling. The K562 cells that were resistant to 10 µM IM exhibited a larger size and higher granularity than their wild-type counterpart (WT), as they showed significantly increased forward and side scatter values determined by flow-cytometry (Fig. 4C, D, and E). Interestingly, TRULI treatment further increased the size and granularity of 10 µM IM-resistant K562 cells, but not wild-type K562 cells (Fig. 4C, D and E). Similarly to wild-type K562 cells, TRULI significantly increased the percentages of apoptotic IM resistant K562 cells (Fig. 4F), and significantly increased the percentage of late-stage erythroid cells derived from IM resistant K562 cells (Fig. 4G). These results suggest that the inhibition of LATS by TRULI increases apoptosis and induces differentiation of IM-resistance K562 cells to late-stage erythroid cells. Furthermore, the effects of TRULI on IM-resistant K562 cells, which express a higher level of LATS, were more pronounced compared to their wild-type counterpart, which had lower levels of LATS1/2. These results suggest that expression of LATS is related to the IM resistance of CML cells and reduction of LATS could sensitize CML cells to imatinib.

### Gene-targeted LATS1 and LATS2 recapitulate the phenotypes of TRULI treated CML cells

**Figure 5:**
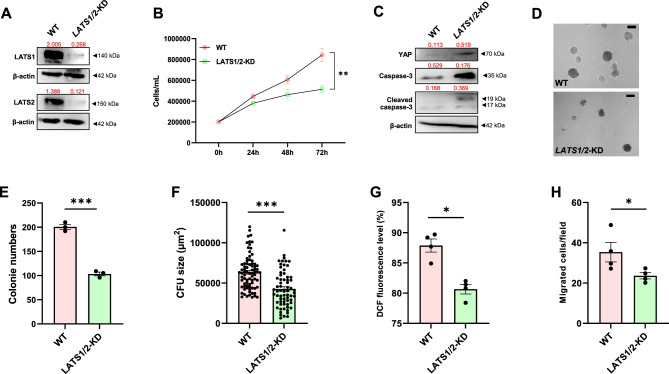
LATS depletion reduced the leukemogenicity of *CML.* Western blot analysis of LATS proteins in shRNA-mediated K562-KD cells (**A**). Cell proliferation count of WT and *LATS1/2*-KD K562 cells (**B**). Protein levels of YAP and caspase-3 in WT- and *LATS1/2*-KD K562 cells (**C**). Representative micrographs of WT and *LATS1 / 2 KD* K562 cells (**D**), and their colony numbers and size (**E** and **F**). Detection of intracellular ROS level in WT and *LATS1/2*-KD K562 cells by the DCFDA / H2DCFDA assay (**G**). Numbers of migrated WT- and *LATS1/2*-KD K562 cells at 48 h in the transwell migration assay (**H**). *p < 0.05; **p < 0.01; ***p < 0.001. Each dot represents the mean of each independent experiment, except that a dot in Fig. 5F represents an individual colony. Band intensities of Western blotting analysis normalized by β-actin were labeled above each lane.

To further confirm the roles of LATS1 and LATS2 in human CML cells, shRNA targeting *LATS1* and *LATS2* was used to generate the LATS1/2—knockdown (KD)—K562 cell line. Western blot showed that the production of LATS1 and LATS2 proteins was successfully abolished in *LATS1/2*—KD—K562 cells (*LATS1/2*-KD) (Fig. 5A). Similarly to TRULI treatment, genetic depletion of *LATS1/2* by shRNA significantly reduced proliferation (Fig. 5B), increased the level of YAP protein, which is a downstream target of LATS, and increased the level of pro-apoptotic cleaved caspase-3 protein in K562 cells (Fig. 5C). Also similar to TRULI treatment, genetic depletion of *LATS1/2* significantly decreased the number and size of K562 colonies in the colony formation assay (Fig. 5D–F), significantly reduced ROS production (Fig. 5G), and significantly reduced the migration ability of K562 cells (Fig. 5H). These data confirm the results obtained from TRULI treatment that inhibition of LATS reduces the tumorigenic properties of CML cells.

### *Reduction of *LATS1*/2 increased the sensitivity of K562 cells to IM treatment*

**Figure 6:**
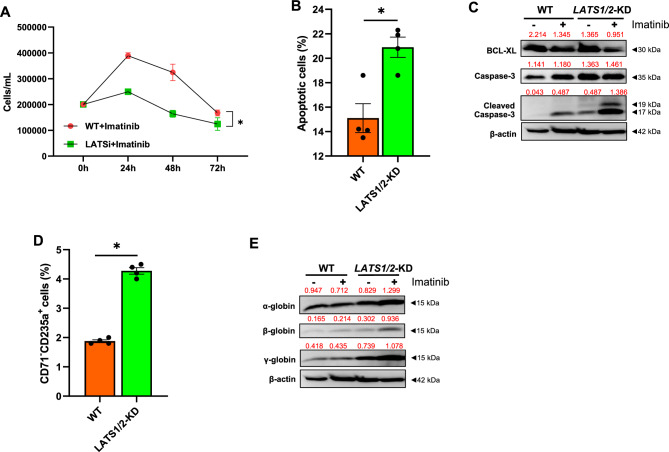
*LATS* reduction enhances IM-induced CML cell death. Cell proliferation of IM-treated WT and *LATS1/2*-KD K562 cells (**A**). Annexin-V^+^ cell death of WT and *LATS1/2*-KD K562 cells treated with IM (**B**). Western blot analysis of BCL-XL and caspase-3 in IM-treated WT and *LATS1/2*-KD K562 cells (**C**). Percentage of late erythroid state in IM-treated WT and *LATS1/2*-KD K562 cells (**D**). Protein levels of human globins in IM-treated WT and *LATS1/2*-KD K562 cells (**E**). *p < 0.05. Each dot represents the mean of each independent experiment. Band intensities of Western blotting analysis normalized by β-actin were labeled above each lane.

To determine the roles of genetic manipulation of *LATS1*/2 in the response of CML to IM treatment, *LATS1/2—KD* cells were treated with IM and the results were compared with those of their WT counterpart. The results showed that *LATS1/2* depletion significantly increased the growth suppressive effect (Fig. 6A) and the apoptotic inducing effect of IM in K562 cells compared to the WT control (Fig. 6B). Consistent with this, the genetic depletion of *LATS1*/*2* increased caspase-3 and cleaved caspase-3 levels compared to the WT control (Fig. 6C). Additionally, IM treatment further increased the level of cleaved caspase-3 protein and reduced the level of the anti-apoptotic protein BCL-XL compared to untreated *LATS1 / 2* KD cells (Fig. 6C). Furthermore, *LATS1/2* depletion also induced a higher level of erythroid differentiation in IM-treated K562 cells compared to WT cells, as demonstrated by the increased number of late erythroid cells and the up-regulation of erythroid-specific marker proteins, α-globin, β-globin and γ-globin (Fig. 6D–E). These results suggest that inhibition of LATS1/2 increased the growth suppressive, apoptotic inducing, and differentiation-enhancing effects of IM in K562 cells.

## Discussion

In 1995, the critical role of the Hippo signaling pathway in controlling cell proliferation and organ size was first identified in *Drosophila melanogaster* through a mosaic genetic screening in which warts (*WTS)*, a component of Hippo signaling, mutation resulted in epithelial tissue overgrowth^6,7^. Since then, aberrant regulation of components of the Hippo pathway has been shown to be associated with the development and progression of many cancers, including hematologic malignancies^15,16^.

The lack of *MST1* kinase, an upstream regulator of LATS1/2, leads to the development of murine lymphoma by inducing chromosomal instability^17^, while down-regulation of *MST1* inhibits the progression of myeloma in humans^18^. Furthermore, YAP and its homolog TAZ, which are targets of LATS1/2 and the main effectors of the Hippo pathways, have been shown to play an important role in both hematopoiesis and leukemogenesis^18,19^. Similarly to MST1, the roles of LATS in hematologic malignancies are still controversial and may depend on the types of cancer. Previous studies show that a decrease in LATS level was associated with an unfavorable outcome in patients with acute lymphoblastic leukemia^20^ and mantle cell lymphoma^21^, suggesting its tumor suppressor role in these cancers. In contrast, an increased level of *LATS2* gene expression was observed in primary CML samples from the chronic and blast crises phases^14^, suggesting that *LATS2* could be involved in the progression of CML. Due to these conflicting results, we therefore investigated the roles of the LATS family in controlling various aspects of CML properties to better understand the roles of the Hippo pathway in human leukemogenesis.

We first characterize the expression levels of the *LATS* family, *LATS1* and *LATS2,* in clinical CML samples. Consistent with a previous finding^14^, we found that the expression levels of *LATS1* and *LATS2*, at the mRNA and protein level, were significantly higher in patients with CML, suggesting their roles in the pathogenesis of CML. We then study the effects of the small molecule inhibitor of LATS1/2 kinase, TRULI, which has been shown to induce proliferation of human and murine cells by increasing YAP expression^22,23^. As expected, TRULI inhibited Hippo signaling by decreasing phosphorylated LATS levels, resulting in a decrease in the levels of phosphorylated YAP and phosphorylated TAZ, which are downstream targets of LATS. It should be noted that while the LATS1 protein decreased to an undetectable level after TRULI treatment, the level of the LATS2 protein was largely unaffected, implying that TRULI might preferentially inhibit LATS1 rather than the LATS2 protein. Our results show that inhibition of LATS activity by TRULI reduced proliferation, decreased clonogenicity, and induced apoptosis of human CML K562 cells^24,25^. Furthermore, TRULI also reduced migration, decreased ROS production, and induced erythroid differentiation of K562 cells. This suggests that the Hippo signaling pathway, especially LATS and its downstream targets, could contribute, at least in part, to promote the tumorigenic properties of CML cells. The effect of LATS on the tumorigenic properties of CML cells was further validated by our shRNA knockdown of the *LATS1* and *LATS2* genes in K562 cells, which showed an effect similar to TRULI treatment.

The main challenge of CML therapy is acquired resistance to IM, a first-line drug for CML treatment, caused by additional mutations in the BCR/ABL fusion gene, resulting in failure of treatment and relapse^26^. Therefore, a novel therapy targeting the Hippo pathway, which our results have shown to regulate several aspects of CML tumorigenicity, could be used to increase the efficacy of IM treatment. In this study, we had established de novo IM-resistant K562 cells to be used as a model representing IM-R patients. Our results show that pharmacological inhibition of LATS1/2 activity significantly induced apoptosis and increased erythroid differentiation of IM-resistant K562 cells. Similarly, depletion of LATS1 and LATS2 proteins in K562 cells by shRNA also increased their levels of apoptosis and erythroid differentiation after IM treatment. These results suggest that inhibition of LATS could increase the response of CML cells to IM by inducing their erythroid differentiation and increasing apoptosis, which is consistent with a previous report showing that LATS1 and LATS2 decrease as cells mature. Based on these results, it is possible that higher levels of LATS1 and LATS2 in our IM-resistant CML patients could contribute to IM resistance by increasing proliferation and reducing apoptosis of CML cells in these patients during IM treatment. Reuven and colleague demonstrated that overexpression of LATS2 inhibited c-Abl activity, reducing the phosphorylation of its targets such as YAP and p73 proteins. LATS2 knockdown triggered DNA damage-induced apoptosis in cells that have permissive DNA damage^27^, which is in line with our findings.

LATS kinase generally acts as a tumor suppressor, several recent publications suggest the distinctive role of LATS in human cancers. For example, up-regulation of LATS was found in CML patients and was associated with advanced stages of the disease^14^. The LATS protein was also up-regulated in nasopharyngeal cancer, and siRNA-targeting *LATS2* inhibited tumor progression^28^. These discrepancies suggest that the function of the Hippo signaling pathway is highly dependent on the type of cancer and can be affected by the tumor microenvironment, such as the ECM component, matrix stiffness, and intra-tumoral fluid pressure.

Although our result contradicts a previous study showing that inhibiting YAP activity by verteporfin further increases apoptosis of CML cells treated with IM^29^. We believe that the role of the Hippo pathway, especially in the pathogenesis of hematologic malignancies, is still controversial. Although YAP has been known to be a direct target of LATS, leading to the assumption that LATS inhibition should have the same effect as YAP inhibition, it is possible that LATS inhibition could affect other protein targets and, by doing so, have a different effect compared to YAP inhibition. Of these, more studies are needed to further characterize the function of LATS, as well as their other downstream targets, to better understand the role of LATS in the regulation of CML cell properties.

Taken together, our results demonstrated that LATS1/2 is involved in the pathogenesis and drug resistance of CML. Targeting LATS could be a promising treatment to enhance the therapeutic effect of a conventional BCR/ABL tyrosine kinase inhibitor such as IM.

## Methods

### Clinical CML specimens

Peripheral blood was obtained from healthy donors and CML patients at the Division of Hematology, Department of Medicine, Faculty of Medicine Siriraj Hospital. All CML samples were collected from newly diagnosed adult CML patients whose leukemic cells contain the Philadelphia chromosome and *the BCR*/*ABL1* gene. The IM-resistant patient is identified by the presentation of *the BCR / ABL1* fusion gene after IM treatment. Mononuclear cells were isolated using Ficoll-density gradient centrifugation. All sample collection processes were carried out after receiving the informed consent from all participants.

### Cell culture, chemicals, and reagents

Human CML cell line, K562, obtained from the American Type Culture Collection (ATCC, Manassas, VA), was cultured in RPMI1640 medium supplemented with 10% (v/v) fetal bovine serum (FBS), 2 mM l-glutamine, 100 U/ml penicillin and 100 μg/ml streptomycin in a humidified incubator with 5% CO_2_ at 37 °C. Cells were seeded into a 12-well plate at an initial plating density of 2 × 10^5^ cells/ml and treated with various concentrations of freshly prepared LATS inhibitor (TRULI, MedChem Express, USA). In some experiments, imatinib mesylate (IM, Sigma-Aldrich, USA) was used to treat cells at a final concentration of 0.2 µM.

### Generation of IM-resistant CML cells

To generate de novo IM-resistant K562 cells, parental K562 cells were continuously exposed to an increasing concentration of IM at a maximum concentration of 10 µM. The resistant cells were collected and their viability was determined by annexin-V/7-AAD staining. The IM response curves on parental cells and resistant cells at 24 h were plotted and the IC50 calculated (Supplementary Fig. 1).

### Knockdown of LATS by short hairpin RNA (shRNA)

pLenti-EmGFP-LATS2/1 KD plasmid targeting *LATS1* and *LATS2* (#52,085, Addgene, USA), which we previously used to generate LATS1 / 2 KD MEG-01 cells^30^, was transfected into HEK293FT packaging cells in conjunction with the VSV-G and dR8.2 plasmid (Addgene, USA) using Lipofectamine 3000 reagent (ThermoFisher Scientific, USA). At 48 h after transfection, viral supernatants were collected, concentrated, and used to transduce K562 cells in the presence of 8 μg/ml hexadimethrine bromide. The transduced K562 cells were then FACS-sorted based on their GFP expression, expanded, and subjected to confirm the expression of the LATS proteins by Western blotting.

### Proliferation assays

Cells were cultured in 96-well plates at a density of 2 × 10^4^ cells/well and treated with various concentrations of TRULI. Cell proliferation was determined by an MTT (3-(4,5-dimethylthiazol-2-yl)-2,5-diphenyltetrazolium bromide) assay. Briefly, cells were incubated with 500 μg/ml of MTT solution (Sigma Aldrich, USA) for 4 h at 37 °C. After incubation, the reaction was terminated by adding a solubilizing reagent and the intensity of the formazan product was measured at 570 nm using a microplate reader (Synergy H1, BioTek, USA). Relative cell growth was calculated by dividing the absorbance of treated cells by that of control cells.

### Migration assay

8 µm-pore size Transwell plates (Corning) were used to assess cell migration. 5 × 10^4^ cells were seeded in 100 µl of low serum medium (2% FBS in RPMI1640 medium) for each condition in the upper well, and 800 µl of complete medium (10% FBS in RPMI1640 medium) was added to the lower chamber. The cells were left to migrate for 48 h at 37 °C. Subsequently, cells that migrate to the lower part were fixed in 4% paraformaldehyde and stained with 5 µg/ml Hoechst 33,342 dye and cell count was performed under a fluorescent microscope.

### Apoptosis and detection of erythroid markers

Cells were washed twice with PBS before incubation with cocktail antibodies consisting of FITC-conjugated anti-CD71, APC-conjugated anti-CD235a, PE-conjugated annexin V and 7-aminoactinomycin D (7-AAD) (all from BD Biosciences, USA) in binding buffer for 15 min at room temperature in the dark. Samples were analyzed by the FACS Canto flow cytometer (BD Biosciences, USA).

### Determination of reactive oxygen species (ROS) level

The level of ROS in cells was determined by cell-permeable reagent 2’,7’-dichlorofluorescein diacetate (DCFDA, Abcam, USA), which is oxidized by ROS to form a fluorescent compound, with excitation and emission spectra of 495 and 529 nm, respectively, according to the manufacturer’s instructions.

### Cell cycle analysis

Cell cycle progression was determined using flow cytometry with Hoechst 33,342 staining. Cells were washed twice with PBS and incubated with 5 µg/ml Hoechst 33,342 in complete medium for 30 min at 37 °C. The cells were then analyzed by the BD Aria Fusion FACS cell sorter (BD Biosciences, USA).

### Clonogenicity assay

3 × 10^2^ K562 cells were cultured in a 12-well plate containing MethoCult™ H4100 medium (STEMCELL Technologies, Canada) with or without TRULI supplementation for 10 days in a humidified atmosphere with 5% CO_2_ at 37 °C. The number and size of the colonies were determined by phase-contrast microscopy.

### Western blot

Fifty micrograms of denatured protein samples were loaded onto 10–15% SDS-PAGE before transfer to 0.45 μm PVDF membranes (Bio-Rad, USA). The transferred membranes were then blocked by incubation with 5% (w/v) skim milk in TBST buffer (25 mM Tris·HCl, pH 7.5, 125 mM NaCl and 0.05% Tween 20) for 1 h at room temperature before further incubation with appropriate primary antibodies (Cell Signaling Technology, USA) for 16 h at 4 °C. The membranes were washed three times with TBST buffer and incubated with appropriate horseradish peroxidase (HRP) labeled secondary antibodies for 1 h at room temperature. The immune complexes were then visualized by immobilon chemiluminescent substrate (EMD Millipore) and digital imager (ImageQuant LAS, GE Healthcare, USA). The list of antibodies and concentrations used in this study is given in Supplementary Table 1.

### RT-qPCR analysis

Total RNA was isolated using TRIzol™ reagent (Invitrogen, USA). cDNA was synthesized from 2 μg of RNA using the RevertAid First Strand cDNA synthesizer kit (Thermo Fisher Scientific, USA). qPCR analysis was performed on a 7500 fast real-time PCR machine (Applied Biosystems, USA) using Power SYBR Green PCR Master Mix (Applied Biosystems, USA). The PCR reaction consisted of SYBR Green PCR master mix, 200 nM forward and reverse primers, and 2 μl cDNA. The total volume was adjusted to 20 μl with nuclease-free water. Initial enzyme activation was carried out at 95 °C for 10 min, followed by 40 cycles of denaturation at 95 °C for 15 s and primer annealing/extension at 60 °C for 1 min. A melting curve analysis was performed to determine the specificity of the primer. The relative expression of each gene was normalized against the level of *GAPDH* of the same sample. The list of primers used in the RT-qPCR analysis is shown in Supplementary Table 2.

### Statistical analysis

Data are presented as means ± SD of at least three independent experiments. Statistical analysis was performed using the non-parametric Kruskal–Wallis test or the one-way ANOVA test, if appropriate. A *p* < 0.05 was considered statistical significance.

### Ethical approval and consent to participate

All sample collection processes were carried out after receiving informed consent from all participants. This study was approved by the Siriraj Institutional Review Board (protocol no. 733/2557 EC1, COA. Si101/2015), Faculty of Medicine Siriraj Hospital, Mahidol University, Bangkok, Thailand. The protocols used in this study complied with the principles set forth in the Declaration of Helsinki, the Belmont Report, the CIOMS Guidelines, and the ICH-GCP. The cell line used in this study was purchased from American Type Culture Collection (ATCC).

### Supplementary Information

Supplementary Information 1.

Supplementary Information 2.

Supplementary Information 3.

Supplementary Information 4.

## Data Availability

The datasets used and/or analyzed during the current study are available from the corresponding author on reasonable request.

## Abbreviations

CML: Chronic myelogenous leukemia
LATS kinase: Large tumor suppressor kinase
IM: Imatinib
YAP: Yes-associated protein
TAZ: WW domain-containing transcription regulator 1
TEAD: Transcriptional enhanced associated domain
KD: Knockdown
WT: Wild-type
