# Supplementary figures and images for: Inhibition of LATS kinases reduces tumorigenicity and increases the sensitivity of human chronic myelogenous leukemia cells to imatinib
Source: Sci Rep. 2024 Feb 18;14:3993. doi: 10.1038/s41598-024-54728-z (PMC10874434; doi:10.1038/s41598-024-54728-z)

# MTT assay of IM-resistant K562 cells

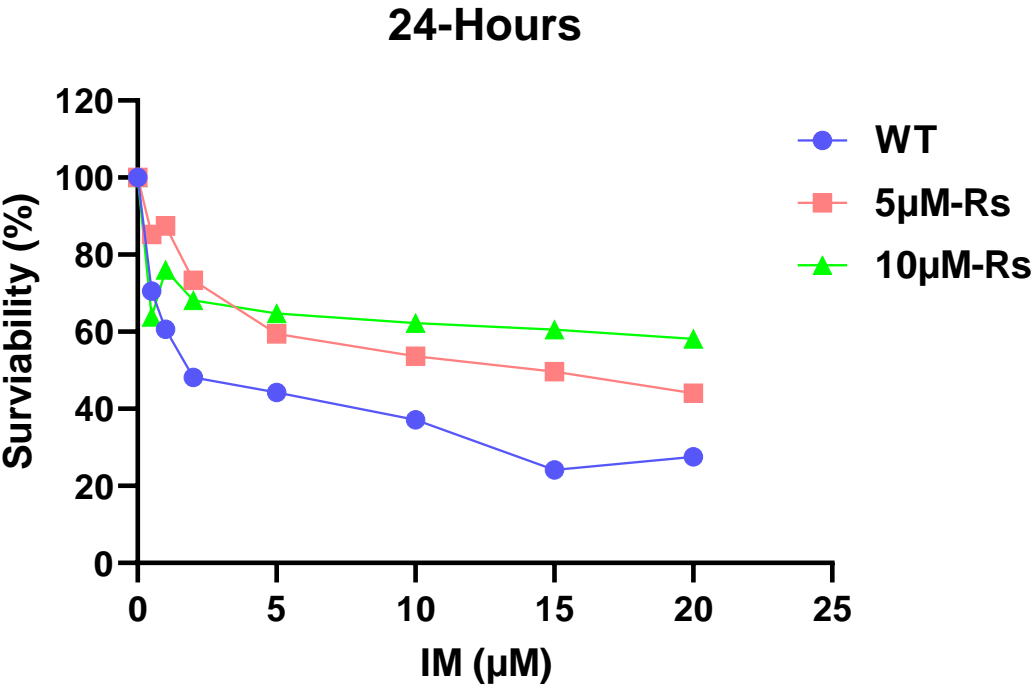

| Cells              | IC50 (μM) at 24 hours |
|--------------------|-----------------------|
| Parental-K562      | 2                     |
| 5 μM-IM resistant  | 15                    |
| 10 μM-IM resistant | 22                    |

Supplement: Supplementary file 1 — Supplementary Information 1. [file 41598_2024_54728_MOESM1_ESM.pdf]
