# Supplementary material for: Inhibition of LATS kinases reduces tumorigenicity and increases the sensitivity of human chronic myelogenous leukemia cells to imatinib
Source: Sci Rep. 2024 Feb 18;14:3993. doi: 10.1038/s41598-024-54728-z (PMC10874434; doi:10.1038/s41598-024-54728-z)
Supplement: Supplementary file 2 — Supplementary Information 2. [file 41598_2024_54728_MOESM2_ESM.docx]

**Supplementary Table 1:** List of antibodies and concentrations used in this study

| **Antibody** | **Dilution** | **Cat. No.** | **Company** |
| --- | --- | --- | --- |
| LATS1 | 1:1,000 | #3477 | Cell Signaling Tech. |
| LATS2 | 1:1,000 | #5888 | Cell Signaling Tech. |
| p-LATS1 (Thr1079) | 1:1,000 | #8654 | Cell Signaling Tech. |
| YAP | 1:1,000 | #12395 | Cell Signaling Tech. |
| p-YAP | 1:1,000 | #13008 | Cell Signaling Tech. |
| BCL-XL | 1:1,000 | #2764 | Cell Signaling Tech. |
| c-MYC | 1:1,000 | #5605 | Cell Signaling Tech. |
| Caspase-3 | 1:1,000 | #9662 | Cell Signaling Tech. |
| α-globin | 1:1,000 | #SC-31110 | Santa Cruz Biotech |
| β-globin | 1:1,000 | #SC-21757 | Santa Cruz Biotech |
| γ-globin | 1:1,000 | #SC-21756 | Santa Cruz Biotech |
| β-actin HRP | 1:10,000 | #A3854 | Merck |
| Goat anti-rabbit HRP | 1:5,000 | #DC03L | Merck |
| Goat anti-mouse HRP | 1:5,000 | #AP124P | Merck |
| Donkey anti-goat HRP | 1:5,000 | #AP180 | Merck |

**Supplementary Table 2:** List of primers used in this study

| **Gene** | **Forward** | **Reverse** |
| --- | --- | --- |
| *LATS1* | GGCACAAACACCATTAGAAACA | AGAAGCTTCAGGACTGAGTTTAGC |
| *LATS2* | AGCAAGAAATGGCCAAAGC | GGTAGAGGATCTTCCGCATCT |
| *GAPDH* | GAAGGTGAAGGTCGGAGTCA | GGGGTCATTGATGGCAACAATA |
