# Supplementary material for: Inhibition of LATS kinases reduces tumorigenicity and increases the sensitivity of human chronic myelogenous leukemia cells to imatinib
Source: Sci Rep. 2024 Feb 18;14:3993. doi: 10.1038/s41598-024-54728-z (PMC10874434; doi:10.1038/s41598-024-54728-z)

Uncropped WB membranes

LATS in CML

Associated with Fig 1

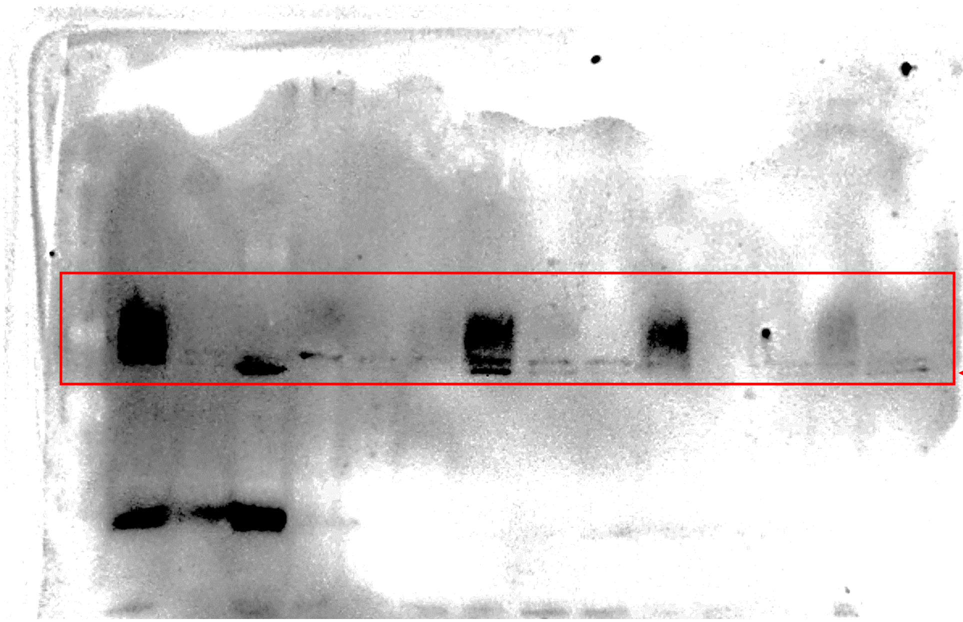

LATS1

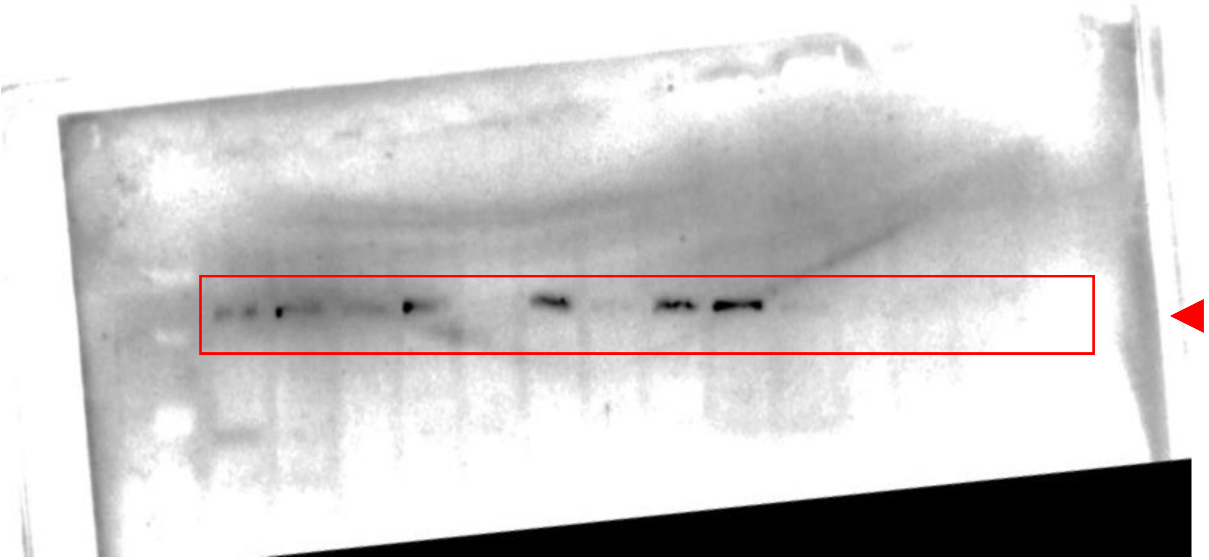

LATS2

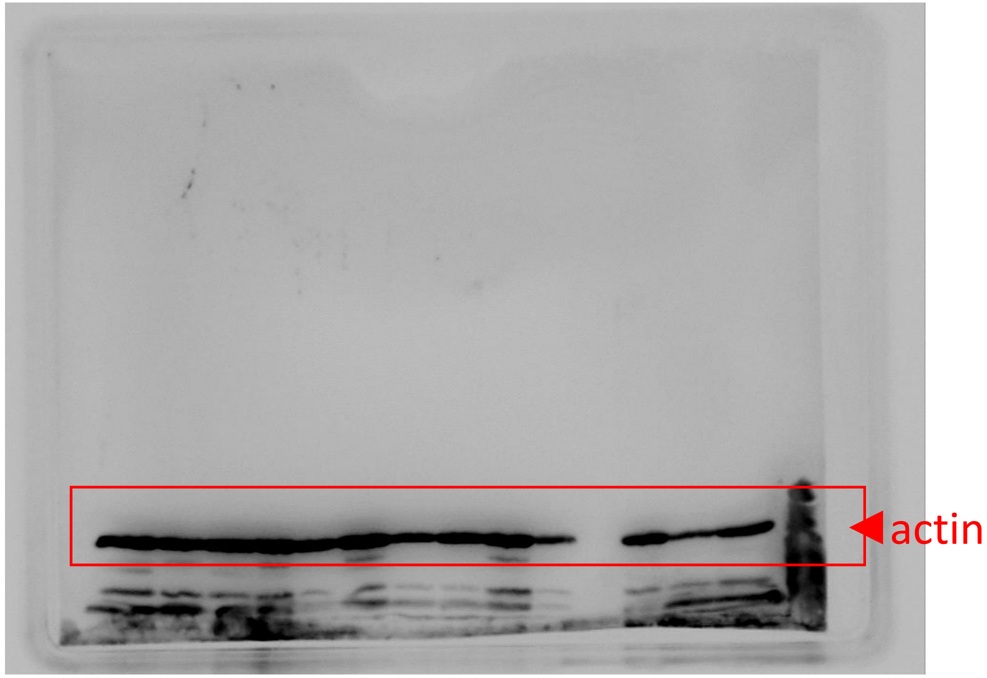

actin

Associated with Fig 2B

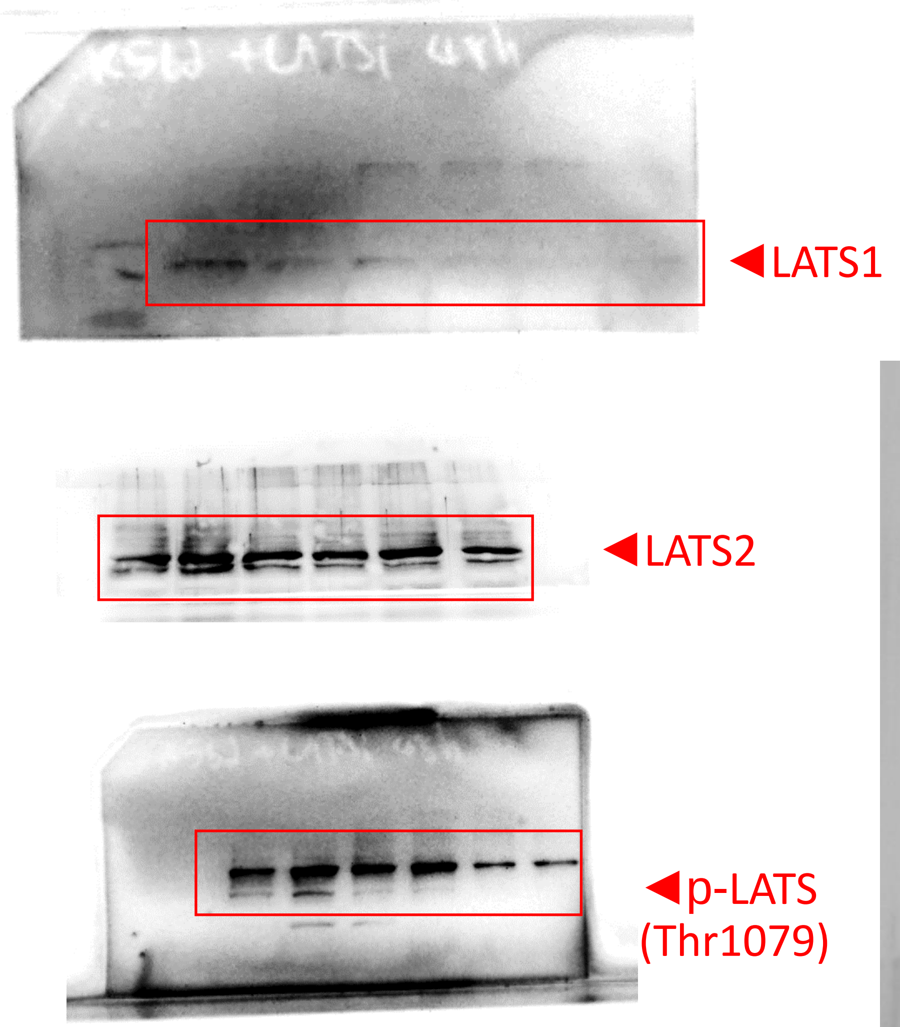

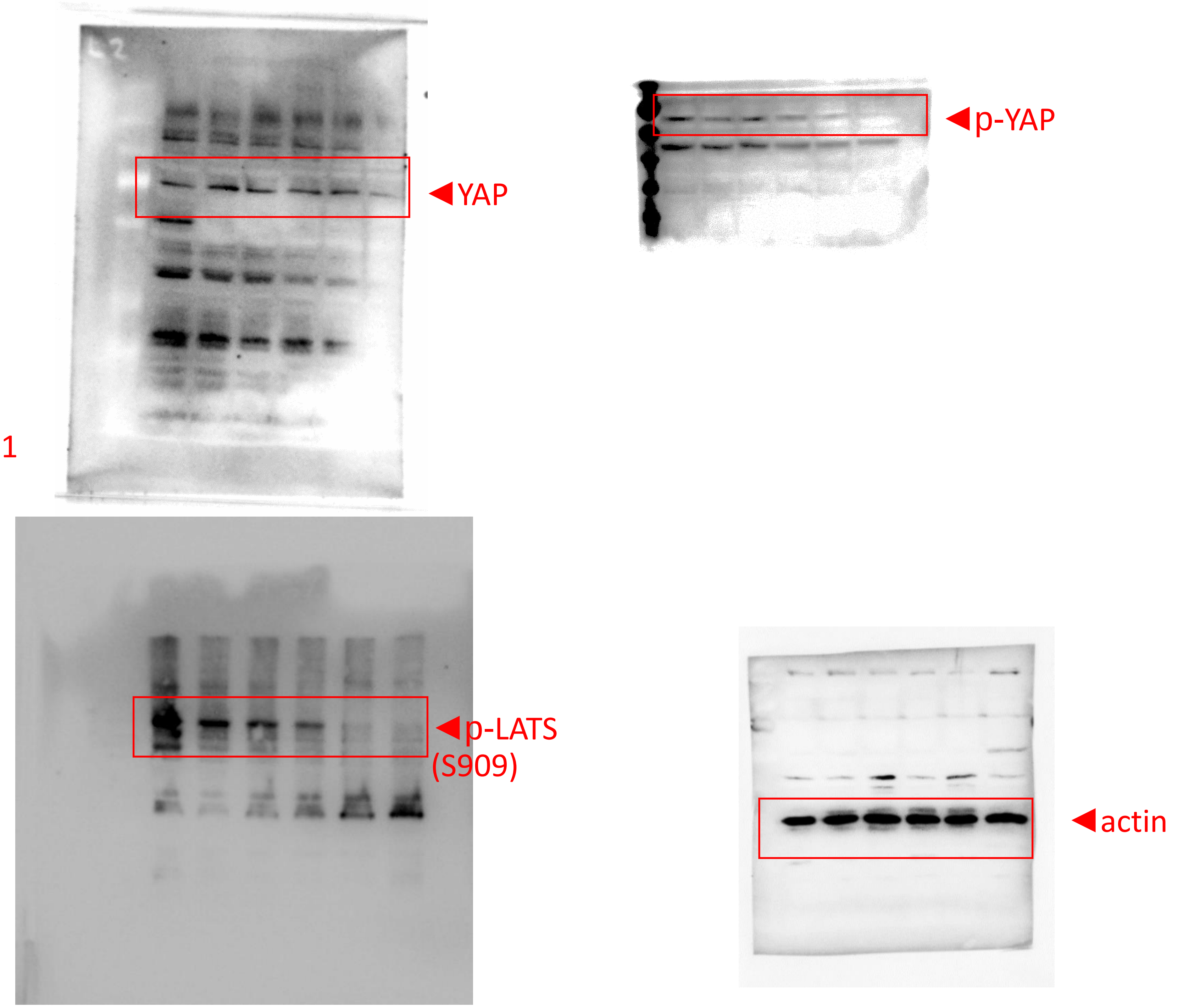

Associated with Fig 2E

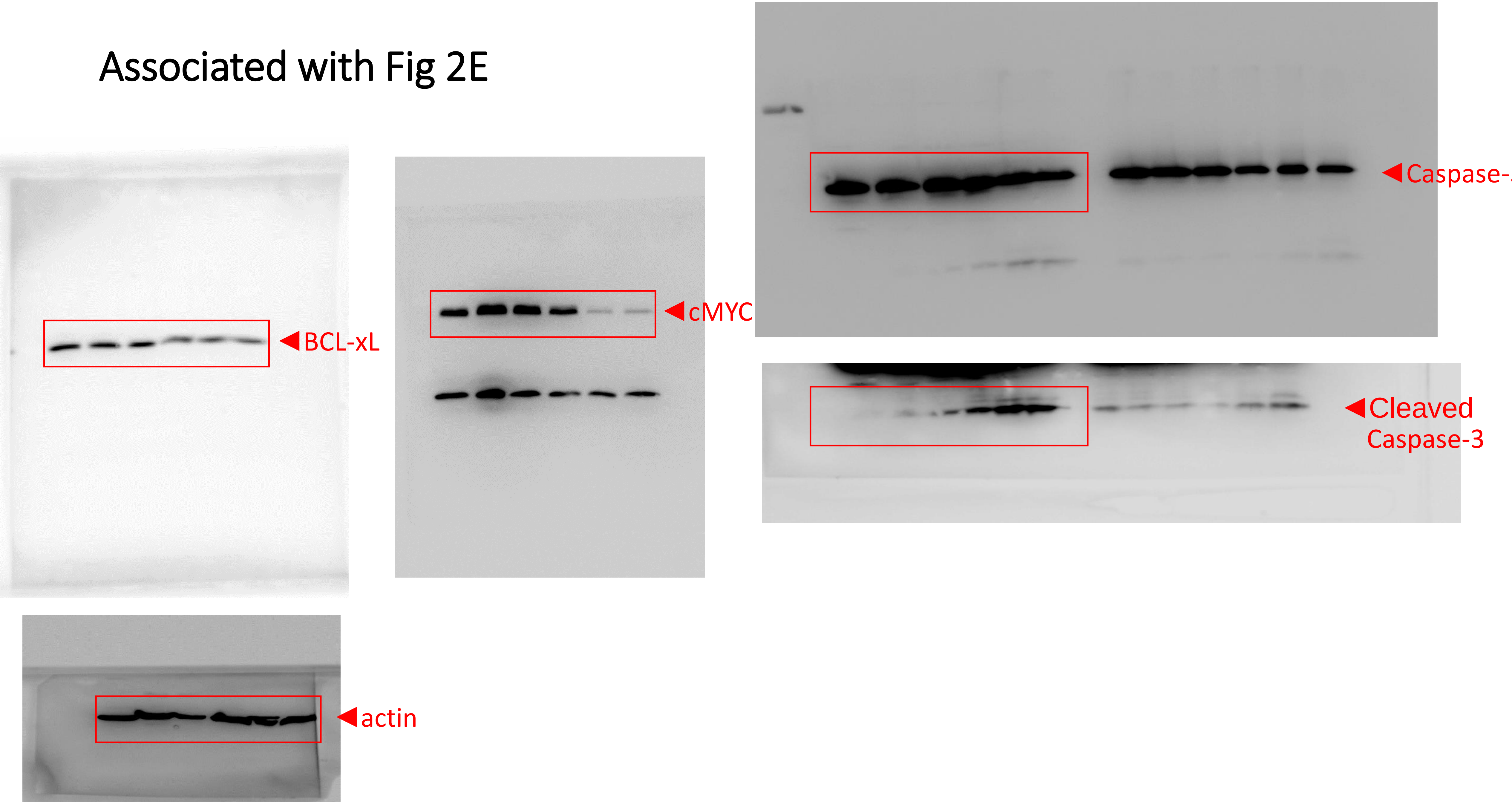

Associated with Fig 2H

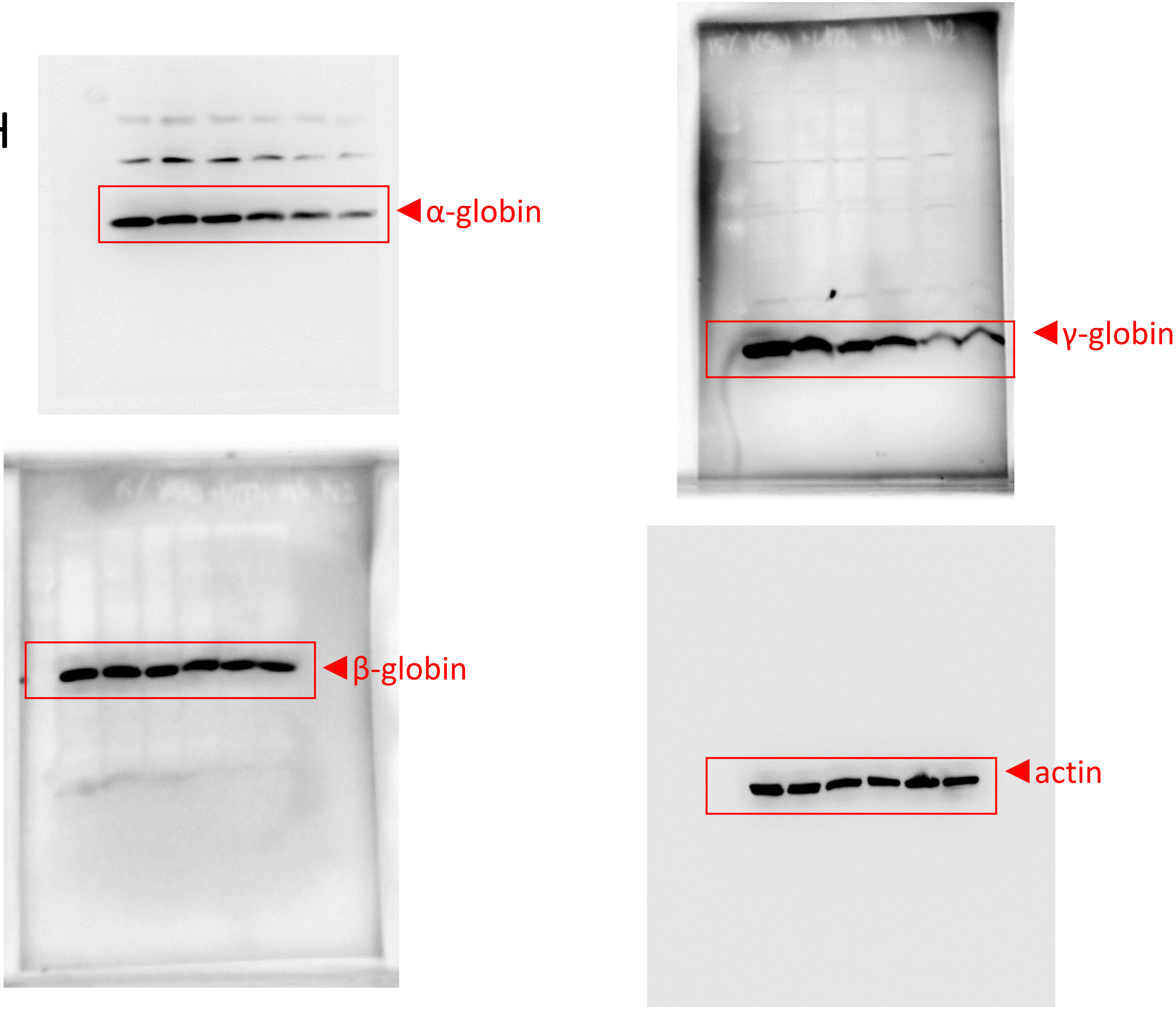

Associated with Fig 4B

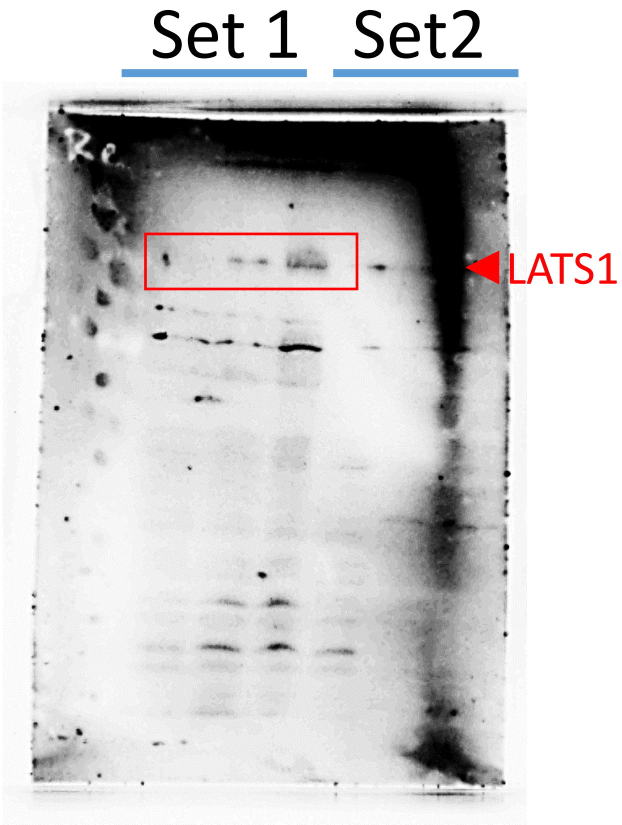

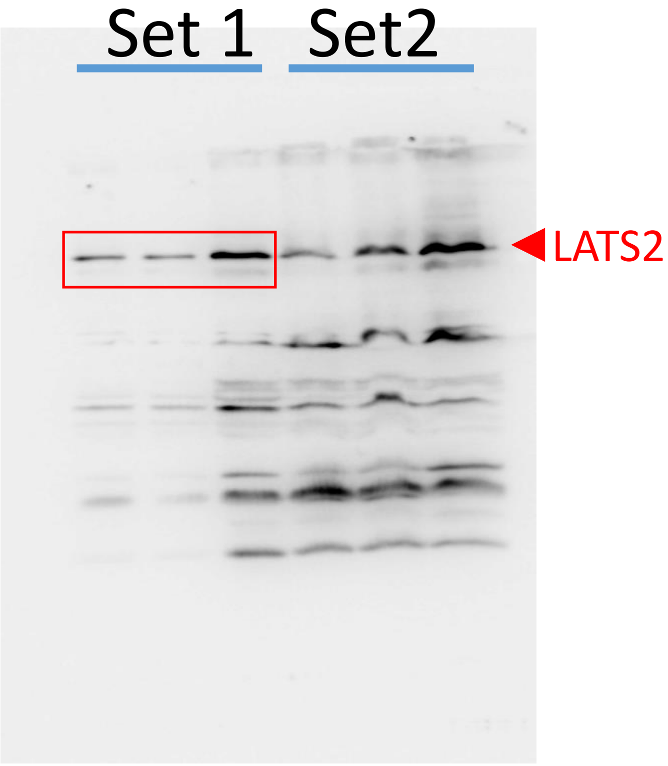

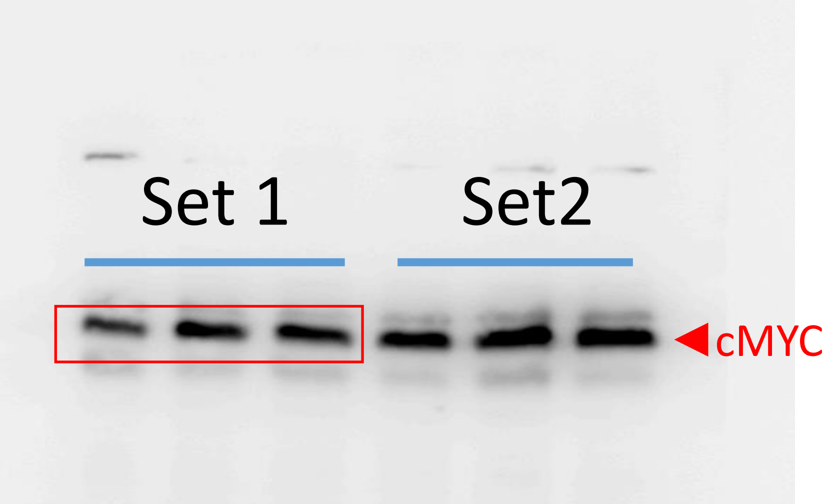

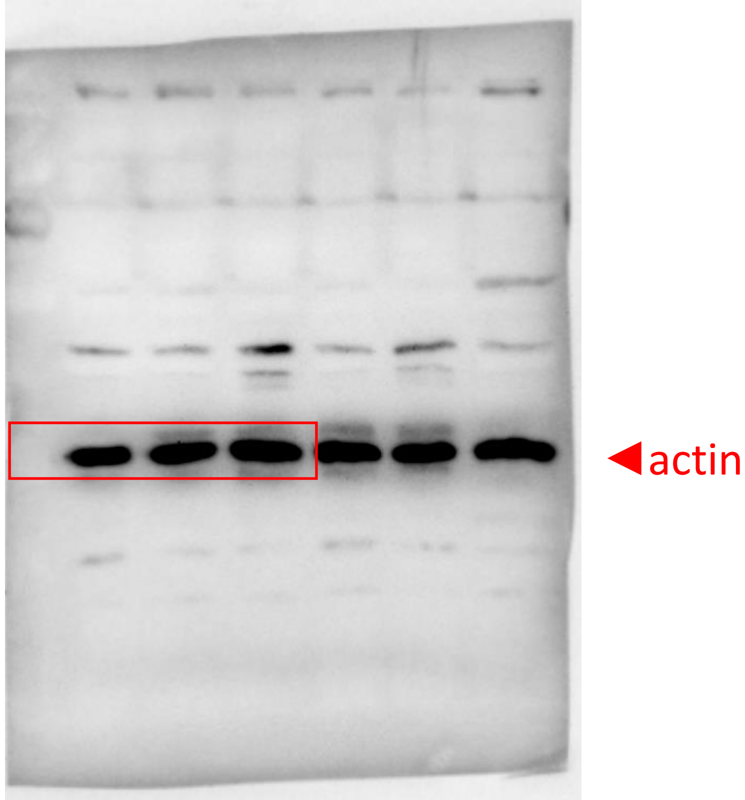

Associated with Fig 5A

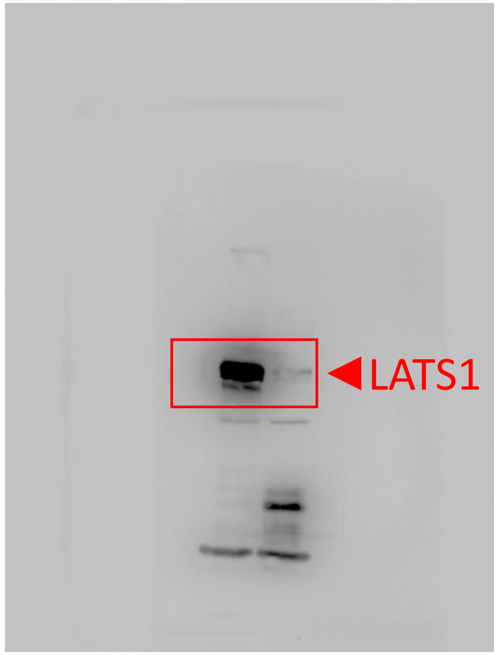

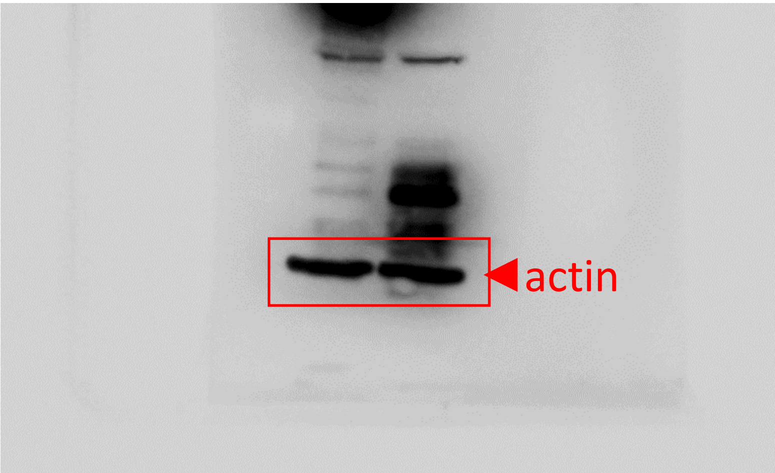

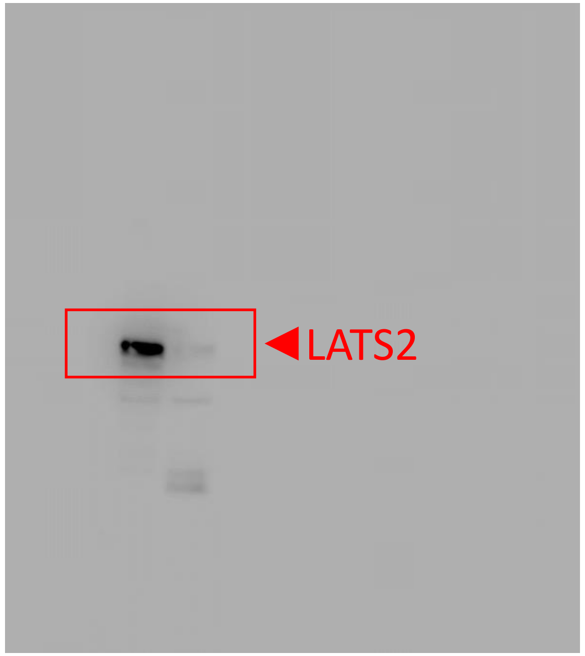

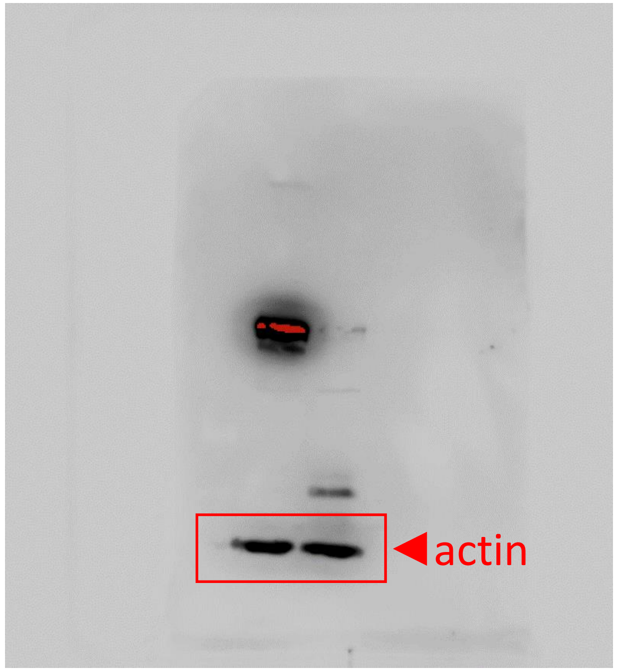

Associated with Fig 5C

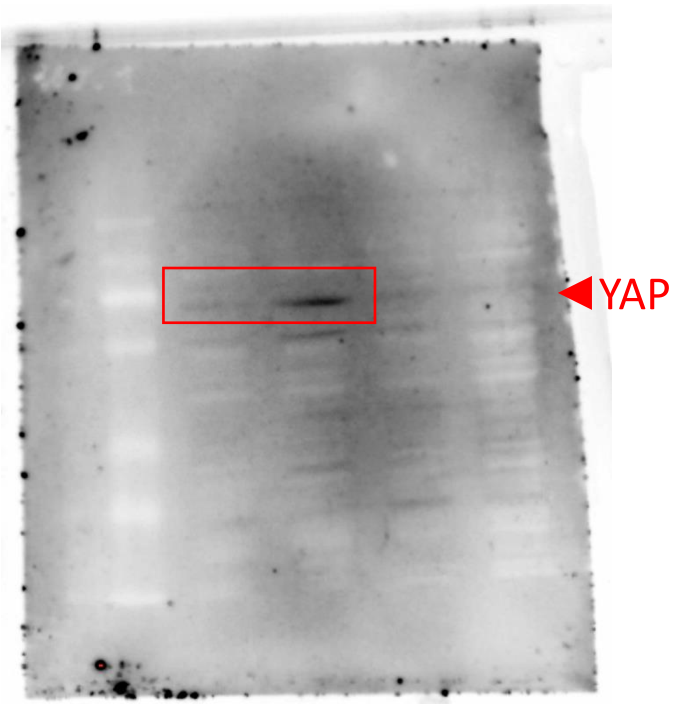

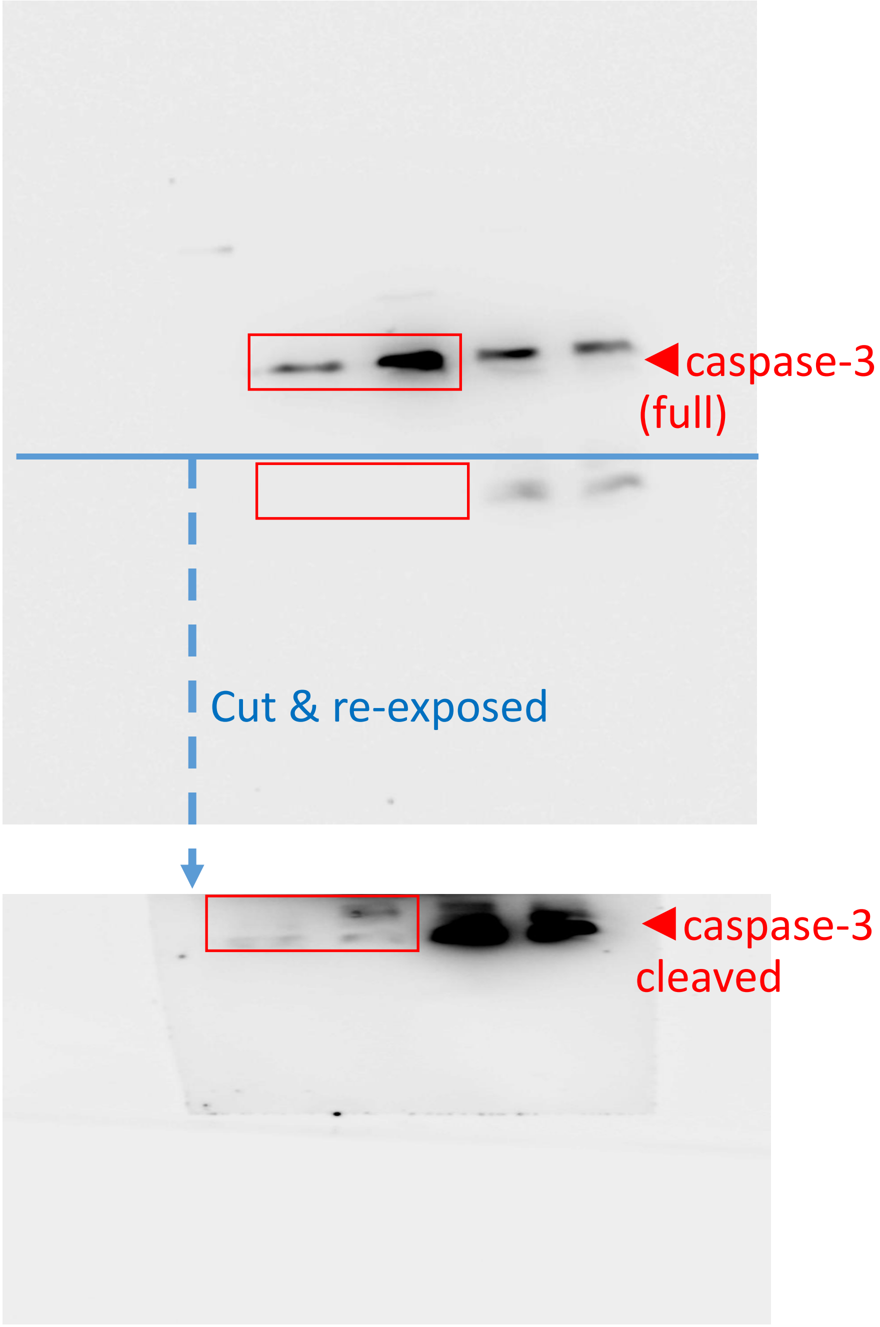

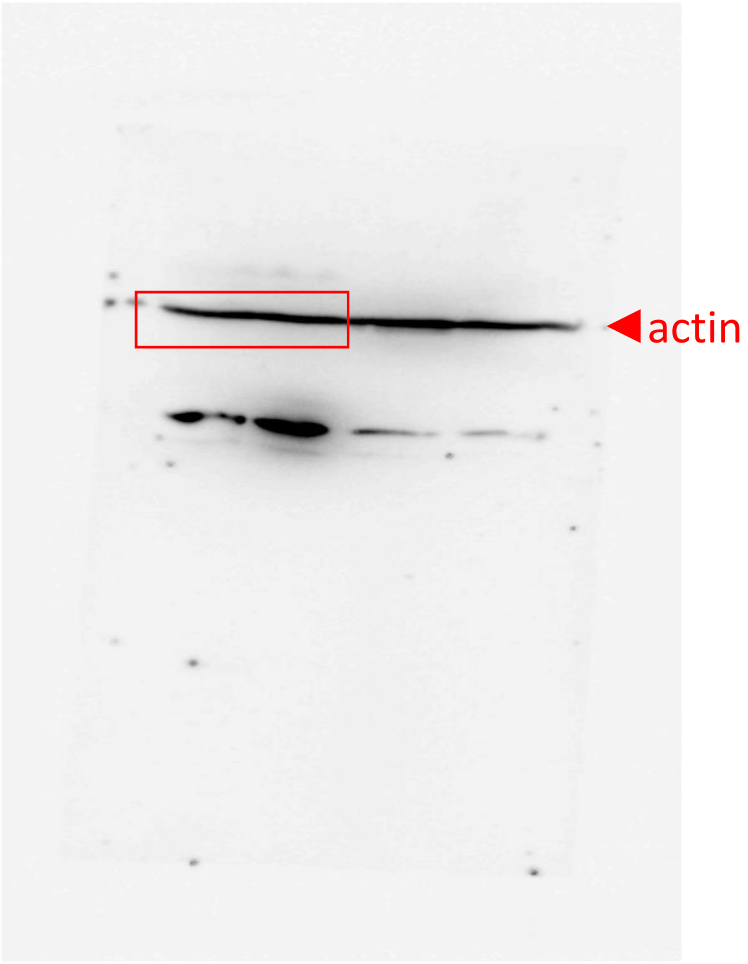

Associated with Fig 6C

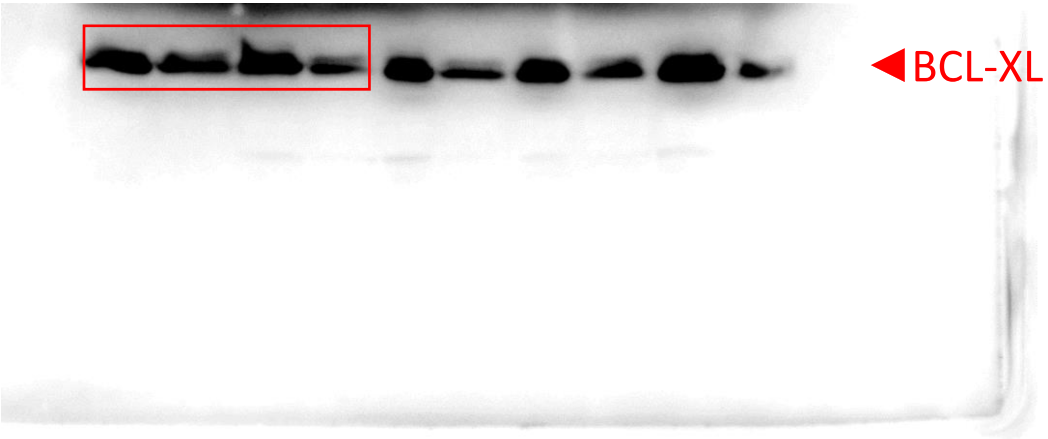

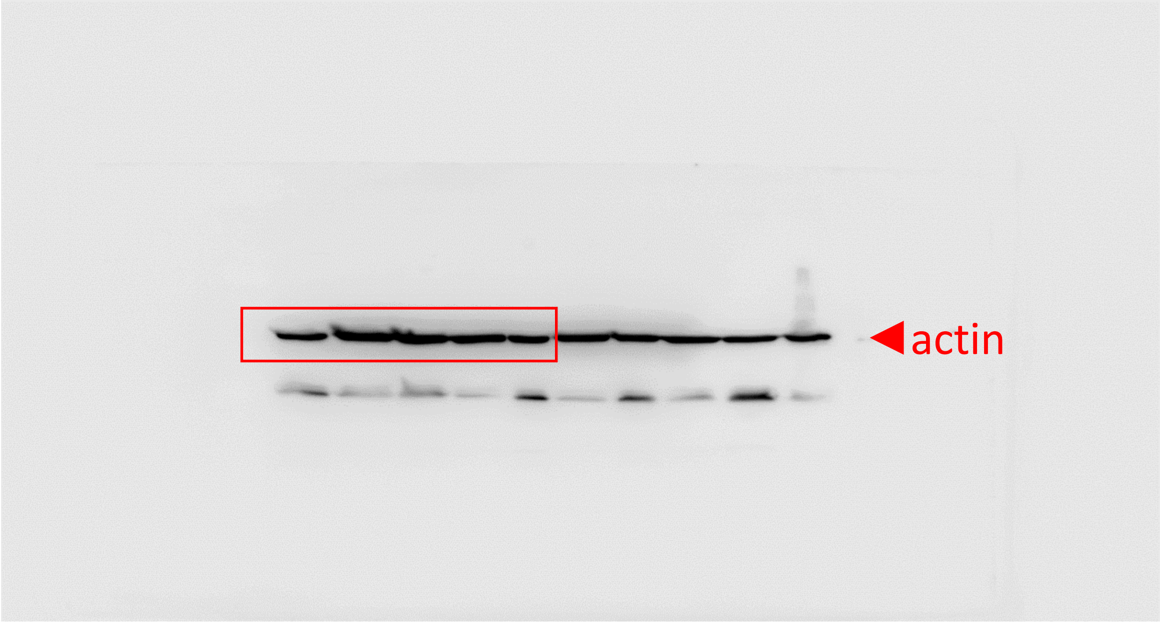

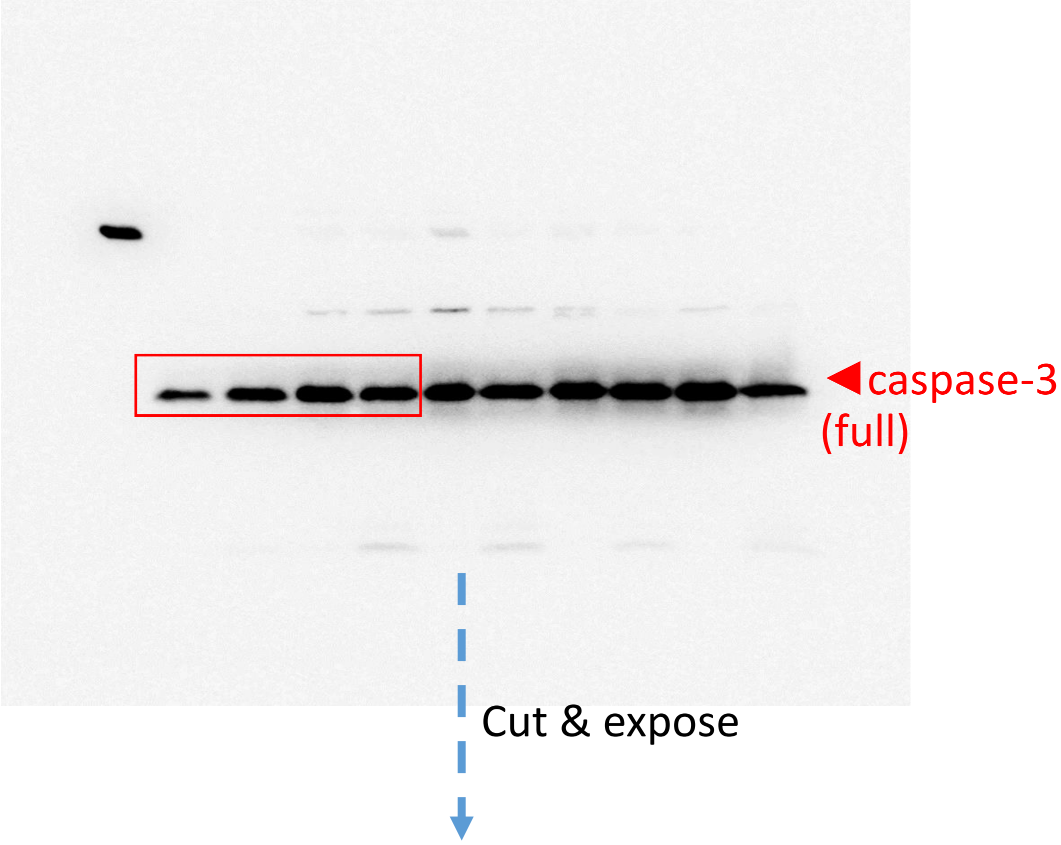

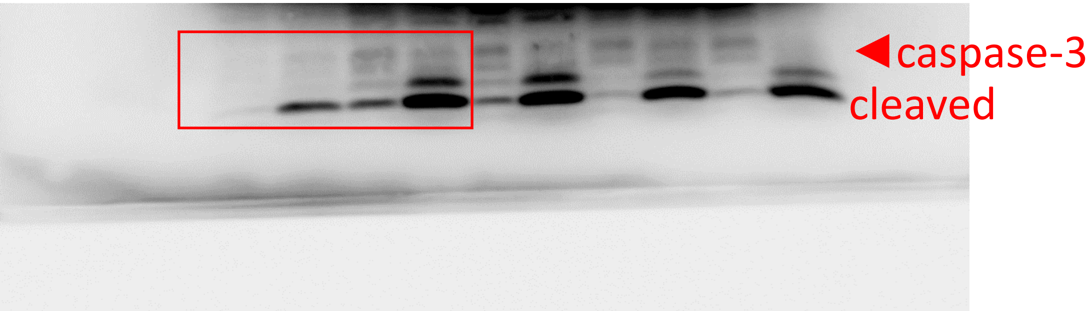

Associated with Fig 6E

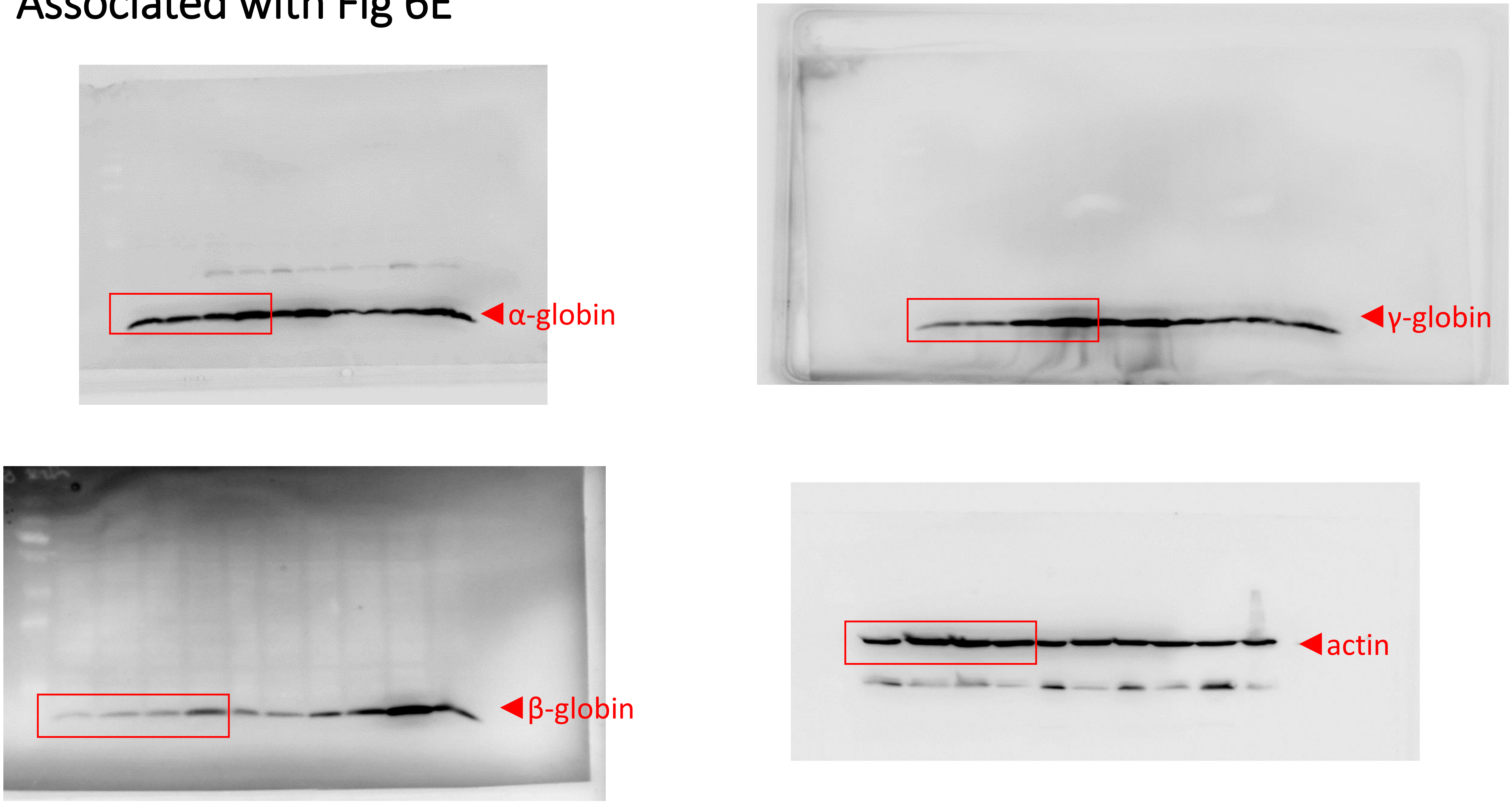

Supplement: Supplementary file 3 — Supplementary Information 3. [file 41598_2024_54728_MOESM3_ESM.pdf]
