# Supplementary material for: Inhibition of LATS kinases reduces tumorigenicity and increases the sensitivity of human chronic myelogenous leukemia cells to imatinib
Source: Sci Rep. 2024 Feb 18;14:3993. doi: 10.1038/s41598-024-54728-z (PMC10874434; doi:10.1038/s41598-024-54728-z)
Supplement: Supplementary file 4 — Supplementary Information 4. [file 41598_2024_54728_MOESM4_ESM.docx]

**Supplementary Figure 1.** MTT assay of IM-resistant K562 cells.
